# Exposure of *Candida parapsilosis* to the silver(I) compound SBC3 induces alterations in the proteome and reduced virulence

**DOI:** 10.1093/mtomcs/mfac046

**Published:** 2022-06-25

**Authors:** Magdalena Piatek, Cillian O'Beirne, Zoe Beato, Matthias Tacke, Kevin Kavanagh

**Affiliations:** Department of Biology, SSPC Pharma Research Centre, Maynooth University, Maynooth, Co. Kildare, Ireland; School of School of Chemistry, University College Dublin, Belfield, Stillorgan, Dublin 4, Ireland; School of School of Chemistry, University College Dublin, Belfield, Stillorgan, Dublin 4, Ireland; School of School of Chemistry, University College Dublin, Belfield, Stillorgan, Dublin 4, Ireland; Department of Biology, SSPC Pharma Research Centre, Maynooth University, Maynooth, Co. Kildare, Ireland

## Abstract

The antimicrobial properties of silver have been exploited for many centuries and continue to gain interest in the fight against antimicrobial drug resistance. The broad-spectrum activity and low toxicity of silver have led to its incorporation into a wide range of novel antimicrobial agents, including *N*-heterocyclic carbene (NHC) complexes. The antimicrobial activity and *in vivo* efficacy of the NHC silver(I) acetate complex SBC3, derived from 1,3-dibenzyl-4,5-diphenylimidazol-2-ylidene (NHC*), have previously been demonstrated, although the mode(s) of action of SBC3 remains to be fully elucidated. Label-free quantitative proteomics was applied to analyse changes in protein abundance in the pathogenic yeast *Candida parapsilosis* in response to SBC3 treatment. An increased abundance of proteins associated with detoxification and drug efflux were indicative of a cell stress response, whilst significant decreases in proteins required for protein and amino acid biosynthesis offer potential insight into the growth-inhibitory mechanisms of SBC3. Guided by the proteomic findings and the prolific biofilm and adherence capabilities of *C. parapsilosis*, our studies have shown the potential of SBC3 in reducing adherence to epithelial cells and biofilm formation and hence decrease fungal virulence.

## Graphical Abstract

**Graphical Abstract:**
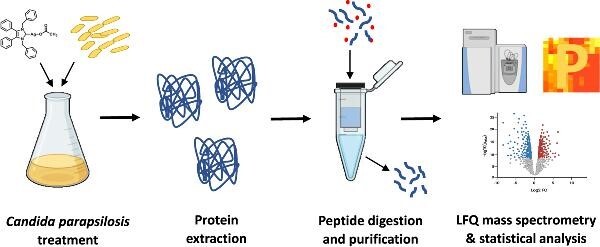
Exposure of Candida parapsilosis to SBC3 induces alterations in the proteome and reduced virulence.

## Introduction

Advances in medicine and technology have improved life quality and reduced mortality rates due to microbial infections over the last 100 years.^1^ Despite this, the incidence of fungal infections is increasing, particularly those infections due to *Candida* spp. and *Aspergillus* spp..^2^ These fungi generally exist as part of the human microbiome, although when left unchecked, they have the capacity to establish infections ranging from superficial mycoses of the skin, hair, and nails to life-threating systemic infections affecting vital organs.^3,4^ Superficial infections are normally easily managed with a fully functioning immune system but can become recalcitrant in immunocompromised individuals and demand potent antifungal therapy.^5^ The increasing rates of fungal infections are largely driven by technological advances such as invasive surgeries and medical equipment, the overuse of antibiotic treatments and by environmental factors such as climate change that provide more favourable conditions to thermotolerant species and/or enhance thermotolerance closer to human body temperature.^6,7^ The survival of infected patients relies on rapid diagnosis and a suitable treatment regimen, although these factors are compromised by prolonged detection methods (with low sensitivity) and the emergence of resistant isolates to first-line treatments.^8^–^10^

The yeast *Candida parapsilosis* is an opportunistic fungal pathogen, and although it is considered less virulent than *Candida albicans*, it has rapidly emerged in recent decades as a serious problem in immunocompromised patients.^11,12^  *Candida parapsilosis* is a commensal of the gastrointestinal tract and skin and is frequently isolated from human hands—a common source of contamination of medical devices where biofilm growth can occur.^11,13,14^ Conventional treatment of this pathogen relies on the use of azole, polyene, or echinocandin antifungal agents; however, the spread of antifungal resistance and limited treatment options have diminished their efficacy.^15,16^ The development of novel, non-toxic antifungal agents with distinct modes of action is urgently required to allow the treatment of resistant isolates.

There has been a reawakening of interest in the antimicrobial properties of silver in recent years.^17,18^ Silver is incorporated into many topical creams, wound dressings, and as medical device coatings in nosocomial settings, while ongoing research seeks to develop novel silver-based drugs with enhanced efficacy and bioavailability.^19^–^21^ Silver exhibits several modes of action, including cell wall rupture to induce cell leakage, facilitation of the generation of reactive oxygen species (ROS), and DNA disruption leading to reduced replication.^22^–^25^ Other mechanisms have been reported, including inhibition of respiration and protein synthesis.^26^–^29^ The antiviral properties of metallodrugs have also been explored, revealing their roles in disrupting the SARS-CoV-2 papain-like protease (PL^pro^). Gold and silver *N*-heterocyclic carbene (NHC) complexes have shown promising inhibitory activity in disrupting this enzyme required for viral replication.^30^

Conjugation of silver to ligands is an effective strategy to improve drug stability and promote more controlled and targeted release of silver.^31,32^ It is essential to ensure a suitable ligand with an adequate level of water solubility, lipophilicity, and stability in normal physiological environments.^33,34^ NHCs constitute a versatile group of organic ligands that readily bind to transition metals.^35^ Structural modifications have produced a library of NHCs with distinct properties; however, a divalent carbon atom bound to nitrogen atom(s) within a heterocycle forms the basis.^33,36^ Guided by the work of Wanzlick and Öfele in the 1960s, Arduengo *et al*. successfully isolated the first stable carbene through the deprotonation of azolium salts in 1991.^37,38^ The ease and efficiency of this new method triggered a wave of NHC research in the development of novel anticancer and antimicrobial agents.^39^–^41^ Numerous studies have shown the broad-spectrum antimicrobial activity of NHC silver complexes for the treatment of gram-positive bacteria [*Enterococcus faecalis, Staphylococcus aureus*, and methicillin-resistant *Staphylococcus aureus* (MRSA)], gram-negative bacteria (*Escherichia coli* and *Pseudomonas aeruginosa*), along with several fungal pathogens (*C. albicans, Candida tropicalis*, and *C. parapsilosis*), among others.^42^–^45^

**Fig. 1:**
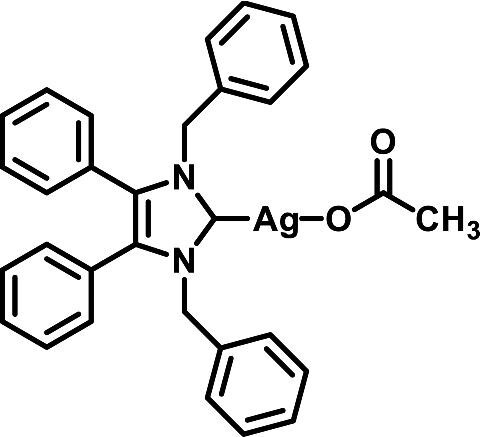
Chemical structure of SBC3.

The NHC 1,3-dibenzyl-4,5-diphenyl-imidazol-2-ylidene silver(I) acetate (SBC3) (Fig. 1), isolated by the Tacke group via Young's synthetic method, has shown excellent inhibitory activity against a selection of resistant bacterial and fungal pathogens, both *in vitro* and *in vivo* using the *Galleria mellonella* insect model.^44,46,47^ SBC3 promoted *G. mellonella* survival against *S. aureus* and *C. albicans* infections, without displaying any adverse effects at the test concentrations, whilst subsequent studies in a murine thigh infection model showed both good tolerability and decreased dissemination of MRSA in response to SBC3 concentrations at the appropriate dosing regimen.^46,47^ The aim of the work presented here was to apply label-free quantitative mass spectrometry (LFQ MS) to *C. parapsilosis* exposed to SBC3 to characterize the proteomic alterations induced by the complex, determine the anti-virulence activity, and thus gain an insight into its antifungal mode(s) of action.

## Results and discussion

### Antifungal evaluation of SBC3 on *C. parapsilosis*

**Fig. 2:**
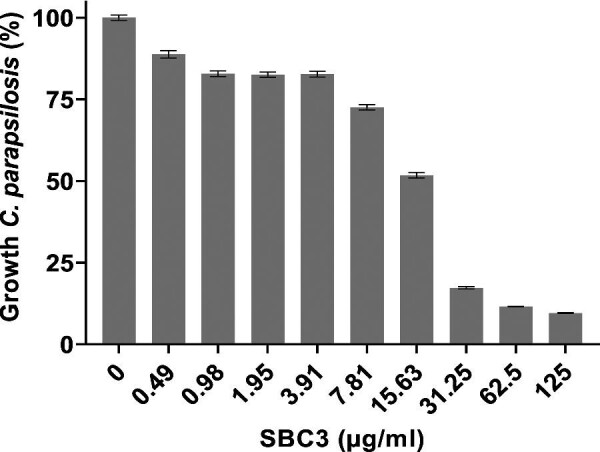
Growth inhibition of *C. parapsilosis* in response to SBC3 treatment. Cells were treated for 24 h at 30°C. All values are the mean ± S.E of three independent experiments, *n* = 24.

SBC3 treatment resulted in up to 90% growth inhibition of *C. parapsilosis in vitro* at the highest test concentration (Fig. 2). The determined MIC_50_ and MIC_80_ values, representing 15 μg/ml and 25 μg/ml, respectively, were selected for subsequent proteomic analysis. The level of growth was also assessed under aerobic conditions (Fig. S1) at these concentrations in accordance with proteomic sample preparation.

### The proteomic response of *C. parapsilosis* to SBC3

*Candida parapsilosis* was exposed to SBC3 (15 and 25 μg/ml) for 8 h and proteins were extracted and purified as described. Purified proteins were identified and quantified via LFQ MS. Statistical analysis confirmed a total of 1281 proteins (post-filtration of contaminants), 451 of which were considered statistically significant and differentially abundant (SSDA) with fold changes ≥1.5.

**Fig. 3:**
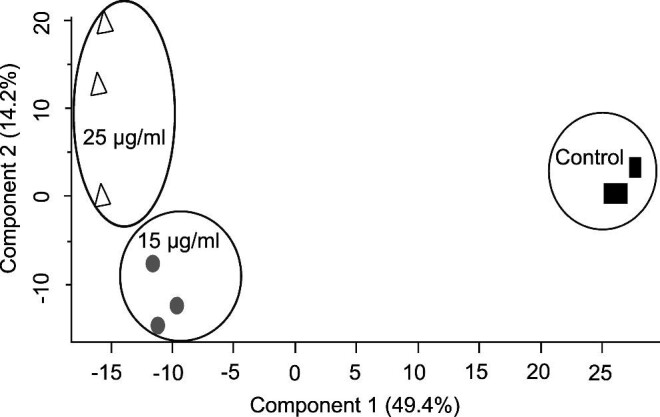
Principal component analysis (PCA) of *C. parapsilosis* proteomes treated with SBC3 (15 and 25 μg/ml) versus control samples (*n* = 3, per sample group).

The entire data set is presented as a principal component analysis (PCA) (Fig. 3), whereby replicate samples are reduced into their corresponding sample groups. The positioning of samples depicts the variability relative to one another, and here, the combined variance between components 1 and 2 amounts to 63.6%. Figure 3 demonstrates distinct differences in the proteomes of *C. parapsilosis* untreated control samples to those challenged with SBC3.

**Fig. 4:**
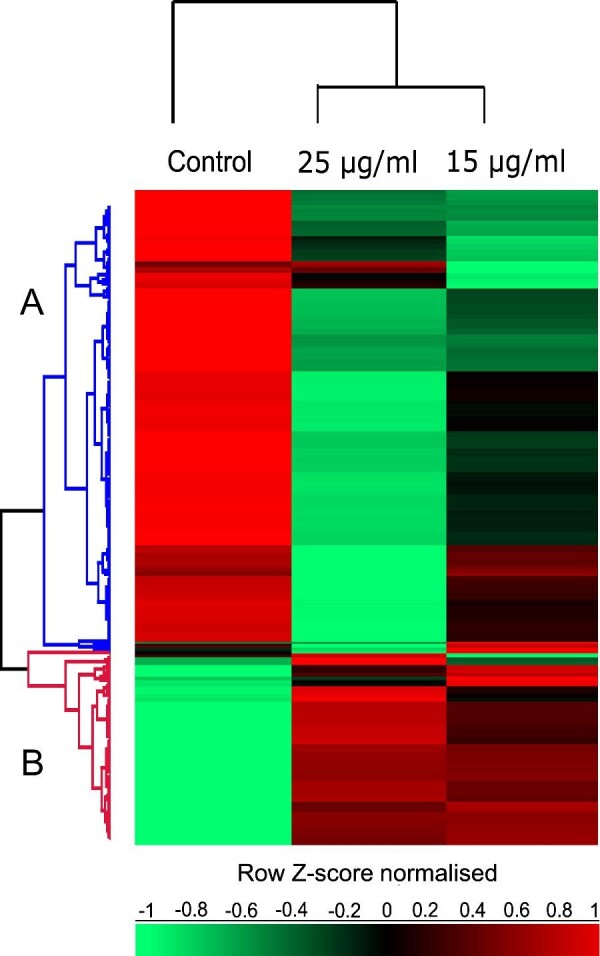
Hierarchal clustering of median expression values of SSDA proteins. Columns comprise of each sample group and rows are clustered (A) and (B), based on similar protein expression profiles, with increased and decreased proteins identified as red and green, respectively. The table includes a selection of statistically enriched GO terms (Benjamini–Hochberg, FDR ≤ 0.05) identified within clusters (A) and (B).

The distinction between treated and untreated samples can be visualized via hierarchal clustering of *z*-score normalized intensity values for 451 SSDA proteins [analysis of variance (ANOVA); Benjamini–Hochberg procedure, false discovery rate (FDR) cut-off of ≤0.05] (Fig. 4). Two row clusters (A and B) were resolved and are based on similar protein abundance trends, with increased and decreased abundance depicted in red and green, respectively. Significant differences in the proteomes of control and SBC3-treated samples are evident. Figure 4 also summarizes the main enrichment gene ontology (GO) terms identified within the clusters, which are predominantly linked with protein synthesis, ribosomal components, and carbohydrate metabolism. All cluster proteins identified are listed in Supplementary File 2.

**Fig. 5:**
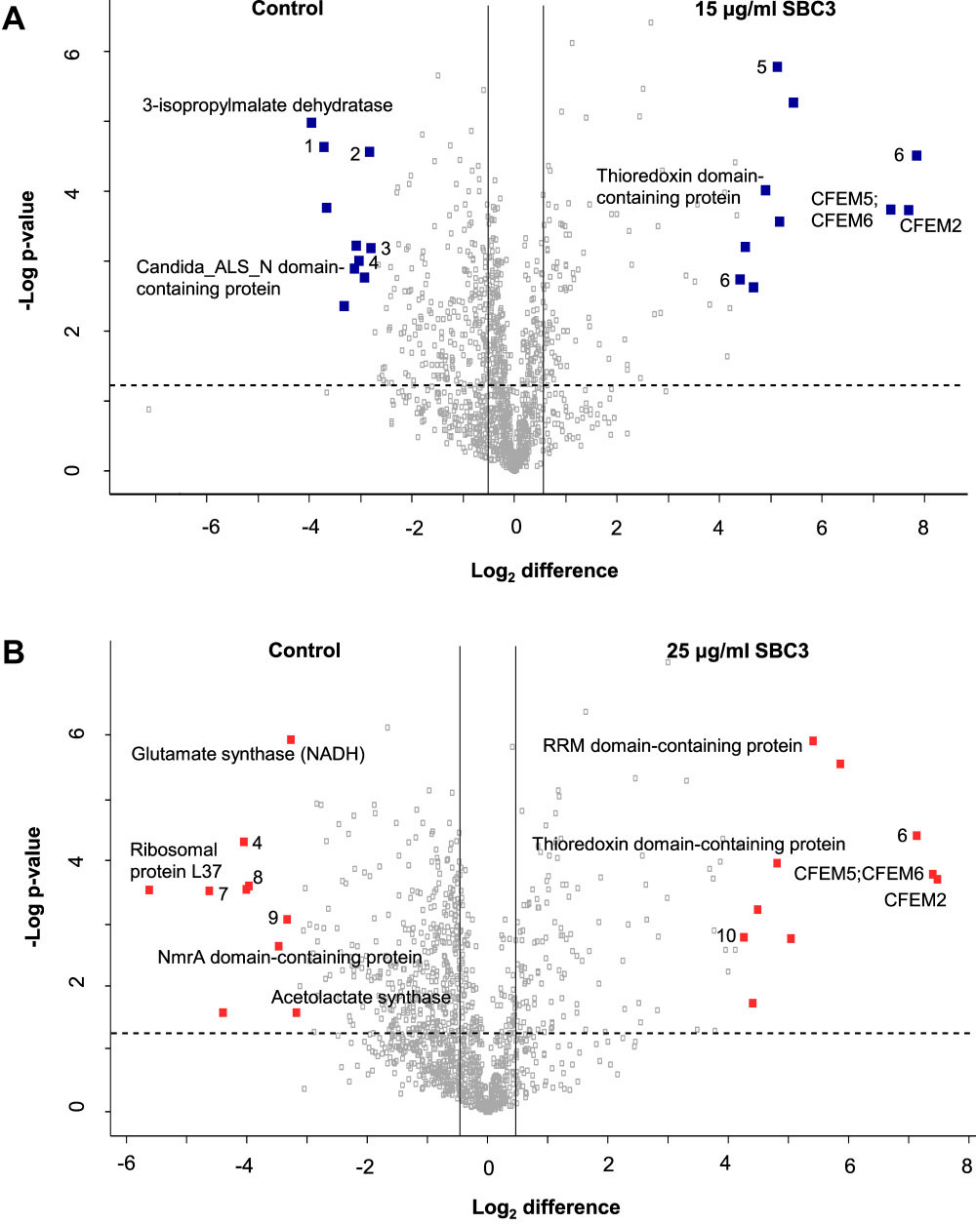
Volcano plots of SSDA proteins in *C. parapsilosis* treated with (A) SBC3 at 15 μg/ml and (B) SBC3 at 25 μg/ml versus untreated control. Pair-wise Student's *t*-tests (*P-*value < 0.05) were used to generate volcano plots with the distribution of quantified proteins according to *P-*value (–log_10_  *P*-value) and fold change (log_2_ mean LFQ intensity difference). Statistically significant proteins (*P-*value < 0.05) lie above the horizontal dashed line, and differentially abundant proteins (fold change ≥1.5) are shown to the right/left of the vertical lines. The top 10 increased/decreased SSDAs are shown in red [unlabelled proteins are uncharacterized; numbered proteins are as follows: (1) glutamate synthase (NADH); (2) cytochrome b5 haeme-binding domain-containing protein; (3) oxidored_q6 domain-containing protein; (4) dihydroxy-acid dehydratase; (5) RRM domain-containing protein; (6) GH18 domain-containing protein; (7) NADH dehydrogenase [ubiquinone] flavoprotein 1, mitochondrial; (8) 3-isopropylmalate dehydratase; (9) zf-CHCC domain-containing protein; and (10) thioredoxin domain-containing protein].

The distribution of all filtered proteins (1281) is represented on volcano plots (Fig. 5), where statistically significant (ANOVA, *P* < 0.05), and differentially abundant proteins (fold change ≥1.5) are located outside of the vertical and horizontal lines. Proteins that increased and decreased in abundance were similar in both treatment groups but different in the extent of their abundance. Among the top 20 proteins most increased in abundance in 15 and 25 μg/ml SBC3 treated *C. parapsilosis*, respectively, were chitinase (+222- and +139-fold), extracellular membrane proteins linked to iron homeostasis, adherence, and virulence common in fungal extracellular membranes (CFEMs) 2 (+207- and 179-fold) and CFEM5/6 (+163- and 172-fold), thioredoxin domain-containing protein (+30- and 28-fold) and uncharacterized protein encoded by candida drug resistance 1 (*CDR1*) gene for xenobiotic detoxification (+25- and 33-fold) (Table S1).

Treatment of *C. parapsilosis* with 15 μg/ml SBC3 decreased the abundance of several proteins associated with amino acid biosynthesis, with the greatest being 3-isopropylmalate dehydratase (–16-fold). Also decreased in abundance were oxidoreductase glutamine amidotransferase type-2 domain-containing protein (–13-fold), an acyltransferase (–10-fold), and Candida_ALS_N domain-containing protein involved in cell adhesion and pathogenicity (–9-fold). In the cells exposed to 25 μg/ml SBC3, there was decreased abundance of ribosomal protein L37 (–49-fold), proteins associated with respiration and electron transport (–25-fold), uncharacterized protein encoded by CPAR2_407940 associated with cellular stress (–8-fold) and squalene monooxygenase used in sterol biosynthesis (–7-fold) (Table S1). These changes are indicative of a high level of cellular stress in response to SBC3. Increased abundance in cell redox and antioxidant proteins is suggestive of coping mechanisms adopted by *C. parapsilosis* to its toxic environment, while decreases in amino acid biosynthesis, ribosomal and respiration proteins, among other virulence traits, provide an indication of the mechanistic roles of SBC3 in inhibiting cell growth and reducing virulence.

### Protein network interaction in response to SBC3

SSDA proteins identified in Perseus were extracted and analysed further using Cytoscape bioinformatics software to map proteins and their associated pathways increased (red) and decreased (blue) in abundance in response to SBC3 treatment. Protein clusters associated with glycolysis/carbohydrate metabolism, ergosterol biosynthesis (also including oxidoreductase activity), components linked with amino acid biosynthesis and the TCA cycle were elevated following treatment (Fig. S2). A considerable number of ribosomal proteins and others associated with protein synthesis (such as transcription, translation, and elongation) were decreased in treated samples relative to controls, in addition to respiration and amino acid biosynthesis proteins. In general, the effects of SBC3 on both treatment groups were comparable, with the higher treatment concentration having a more pronounced effect on the differential abundance of proteins.

### The effect of SBC3 on cellular respiration

**Fig. 6:**
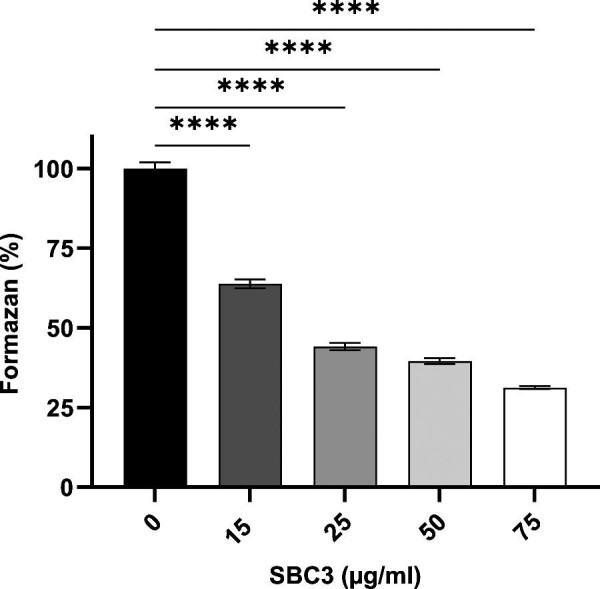
Respiratory activity of *C. parapsilosis* treated with SBC3 for 12 h. Statistically significant reductions in respiration are shown by decreased formazan production in SBC3-treated cells. All values are the mean ± S.E of three independent experiments, *n* = 48 (****P < 0.0001).

Reduction in respiration in response to SBC3 was ascertained via a 2,3,5-triphenyltetrazolium chloride (TTC) test whereby dehydrogenase enzymes in the mitochondria of viable cells have the capacity to reduce colourless TTC to red formazan crystals in growth media. The calorimetric change is directly proportional to the rate of respiratory activity in cells. The results in Fig. 6 substantiate proteomics findings, showing a 36–69% reduction in respiration following exposure to SBC3 at 15–75 μg/ml.

### Assessment of the effects of SBC3 on *C. parapsilosis* adherence and biofilm formation

Proteomic analysis of cells exposed to SBC3 indicated alterations in the abundance of proteins associated with cell wall synthesis (chitinases), adherence, and biofilm formation (Candida_ALS_N domain-containing protein, CFEM2 and 5/6) (Fig. 5, Supplementary File 1), and ergosterol biosynthesis (encoded by *ERG4, ERG11*, and *NCP1*) (Fig. S2).

**Fig. 7:**
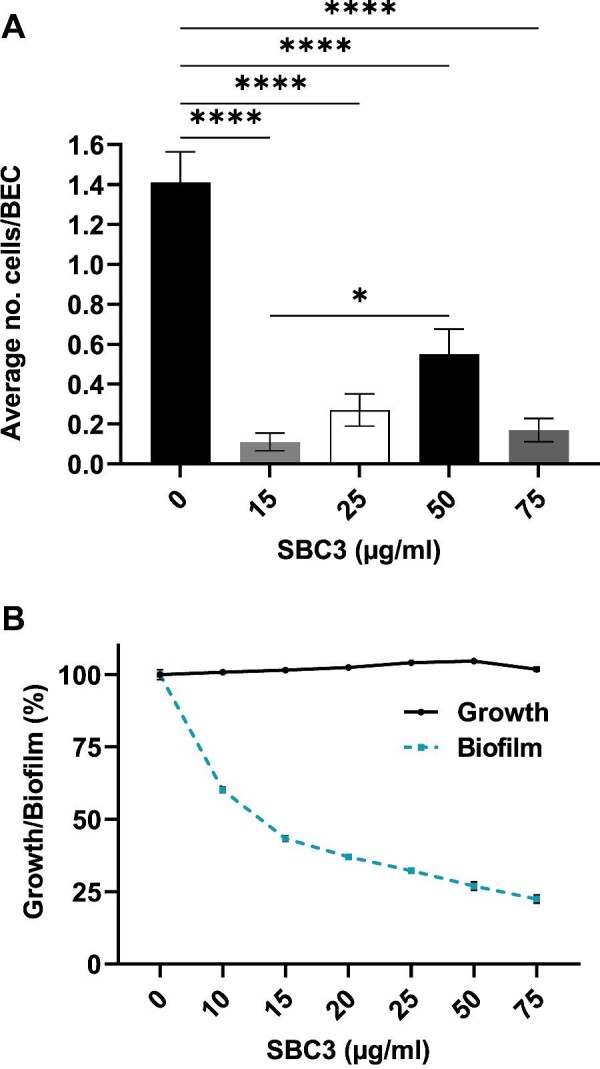
The anti-adherence and anti-biofilm effects of SBC3 on *C. parapsilosis*. (A) Pre-treatment of *C. parapsilosis* with SBC3 significantly reduced the number of adhered yeast cells on BECs, compared to control samples. All values are the mean ± S.E., *n* = 100 (****P < 0.0001). (B) Reduction in biofilm in response to SBC3 after 48 h. Growth remains unaffected by all treatment concentrations. All values are the mean ± S.E., *n* = 32.

The effects of SBC3 on adherence of *C. parapsilosis* to buccal epithelial cells (BECs) and biofilm formation on an abiotic surface were characterized to ascertain whether the proteomic alterations had an impact on the cell's disease-causing potential. *Candida parapsilosis* grown with SBC3 for 24 h was isolated and co-incubated with BECs for 120 min to permit the attachment of cells. Adhered yeast cells were enumerated on each BEC for each treatment group, revealing pre-treatment with SBC3 (15, 25, 50, and 75 μg/ml) resulted in a 76.6 ± 15.5% reduction in adherence (Fig. 7A).

To assess the effect of SBC3 on biofilm formation, *C. parapsilosis* cells were cultured in the presence of SBC3 (10, 15, 20, 25, 50, and 75 μg/ml) for 48 h. The growth of cells was initially measured to verify biofilm reduction was a direct result of SBC3 treatment rather than reduced cell presence. Post-removal of planktonic cells, the remaining adhered cells were stained and measured spectrophotometrically. The results showed a dose-dependent reduction in biofilm formation by 40–76%, while no reduction in cell growth was observed for all test concentrations (Fig. 7B).

**Fig. 8:**
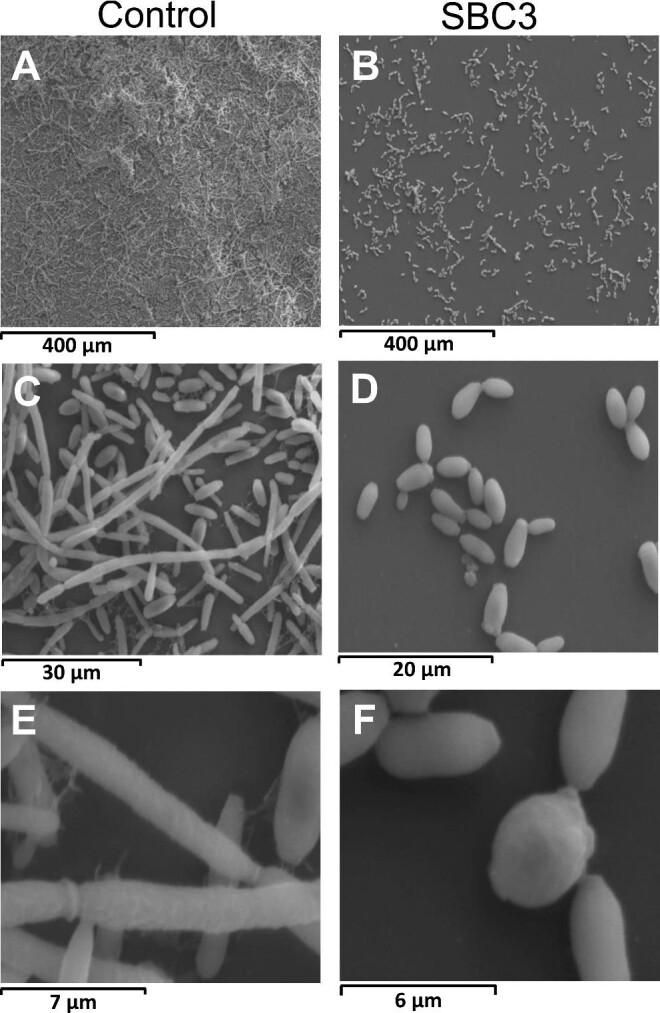
SEM images of *C. parapsilosis* grown in the absence (A, C, E) and presence (B, D, F) of SBC3 for 48 h. Various magnification levels exhibit a reduction in biofilm and altered morphologies in treated samples.

SEM was employed to view structural and morphological changes in *C. parapsilosis*, further supporting the antibiofilm capabilities of SBC3 (Fig. 8). A dense network of cells in both yeast (oval and singular) and pseudohyphal (elongated and attached) forms was evident in untreated control samples (Fig. 8A and C). Filamentous cells layered on top of aggregated cells in the yeast form are adhered to the slide surface, aided by extracellular matrix (ECM) material (Fig. 8E). To observe the full potential of the complex, the highest test dose of SBC3 (75 μg/ml) was selected to treat cells. As a result, cells appeared more dispersed, with a reduced presence of biofilm (and ECM material) (Fig. 8B) and were predominantly in the yeast form with a rounded morphology (Fig. 8D) and rough texture characteristic of cell wall damage shown in Fig. 8F.^17^

## Discussion

While *C. albicans* remains a significant fungal pathogen, the epidemiology of *Candida* infections has altered over recent years with increasing reports of *C. parapsilosis* infections.^11^ Incident rates are likely to increase with the ongoing use of aggressive immunosuppressive therapies and invasive medical procedures, despite limited treatment options.^48^ Much of *C. parapsilosis* virulence is due to its adherence ability and biofilm formation.^49^

Silver research has largely focused on the antibacterial activity of silver despite demonstrating promising antifungal properties. The activity of silver against bacteria and fungi appears largely universal, having the capacity to induce ROS production. During normal cell activity, viable cells produce low levels of ROS in the mitochondria that are modulated with antioxidants. Silver treatment can perturb this homeostasis via the binding of enzymes in the respiratory chain, resulting in oxidative damage to nucleic acids, proteins, and lipids. Silver can also contribute to structural damage. Association with cell membranes/walls and formation of pores alters permeability, resulting in ion leakage, and ultimately, cell death.^50^–^53^ Studies have also reported the inhibition of filamentation, and hence biofilm formation, in fungi, while Kim *et al*. demonstrated inhibition of growth via cell cycle arrest and prevention of cell budding in *C. albicans* in response to silver.^17,22^

Quantitative label-free proteomics were employed to assess the effect of SBC3 on the proteome of SBC3-treated *C. parapsilosis* cells and identified considerable increases in the abundance of chitinase. Chitin is a polysaccharide component of the fungal cell wall that provides structural stability to the cell.^54^ Cell growth relies on an interplay between biosynthetic chitin enzymes and chitin degrading enzymes (chitinases) for morphological changes and cell turnover.^55,56^ SBC3 induced an increased abundance of chitinase, encoded by *CPAR2_502120*/*CPAR2_502130*. In addition, several CFEM proteins (2, 5–7) were significantly more abundant in both SBC3-treated sample groups. The CFEM domain comprises eight conserved cysteine residues belonging to a group of proteins specific to fungi.^57^ CFEM proteins function in adherence, biofilm formation, and pathogenicity, with other suspected functions as signal transducers and cell-surface receptors.^58,59^ It is believed that CFEM proteins influence biofilm formation in *C. parapsilosis*, although their exact effect has not been fully elucidated.^60^ We hypothesize the elevated levels of the aforementioned proteins are a compensatory mechanism to withstand the effects of SBC3 in the disruption of cell wall integrity and biofilm formation.

ATP-binding cassette (ABC) transporter proteins act as efflux pumps for xenobiotics, drugs, and metals, and are encoded by *CDR1* and *CDR2* genes.^61,62^ Resistance typically coincides with altered expression of multiple genes, where *C. parapsilosis* showed reduced susceptibility to fluconazole from increased expression of *CDR1* and *ERG11*.^63^ SBC3 reduced expression of the ergosterol synthesis gene *ERG11* while *ERG4* was increased in treated samples in combination with *NCP1*-encoded NADPH cytochrome P450 reductase required for cell detoxification.^64^ The link between increased *ERG4* and azole resistance remains, unclear although significantly increased levels of ERG4 mRNA have previously been detected in azole resistant *C. albicans* compared to susceptible isolates.^65^ Ergosterol is a lipid component of biological membranes, which maintains structure and permeability of the cell and therefore, these alterations in the cell membrane may possibly reveal a method of cellular detoxification and physical defense against environmental stress.^66,67^

Evidence of cellular stress response is further revealed with an increased abundance of oxidoreductases. Thioredoxin plays a crucial role in detoxifying peroxides and the flavoenzyme thioredoxin reductase catalyses this process to maintain cell redox homeostasis and protect against oxidative stress.^68,69^ While studies have presented possibilities of thioredoxin reductases as antifungal targets, sub-inhibitory doses of SBC3 significantly elevated levels of thioredoxin domain-containing protein.^68,70,71^

The most apparent change in SBC3-treated proteomes was the decrease in the abundance of proteins associated with protein synthesis. A large proportion of protein clusters generated on Cytoscape (Fig. S2) contain translation/elongation factors, tRNA synthetases, ribosomal constituents, along with ribosome biogenesis, RNA binding, and rRNA processing proteins. Ribosomes are complex macromolecules that facilitate amino acid linkage to generate peptide chains, and the assembly of ribosomes is an intricate process of rRNA transcription, folding, and coupling of 60S and 40S subunits.^72^–^74^ Ribosomal protein L37 contributes to early ribosome assembly and demonstrated the greatest change (–49-fold) among decreased proteins in *C. parapsilosis* exposed to 25 μg/ml SBC3.^75^ Ribosome assembly, and indeed protein synthesis, are essential to sustain normal cell growth, and this vulnerability has been exploited in novel and existing drug targets. Sun *et al*. suggested a similar mode of action for a repurposed formulation of a thiosemicarbazone via transcriptional analysis.^76^ A novel sordarin derivative has shown specific action against the protein synthesis elongation cycle in some *Candida* spp. and bears no adverse effect on mammalian protein synthesis.^77^

Amino acids are integral components of proteins, and synthesis and metabolism require strict control for adequate protein formation and nutrient acquisition.^78,79^ In the yeast *Saccharomyces cerevisiae*, amino acid production relies on feedback regulation of biosynthetic enzymes encoded by *ILV* genes.^78^ The data presented here identified decreased expression of these genes listed as follows with their protein products: *ILV1* (threonine dehydratase), *ILV2* (acetolactate synthase), *ILV3* (dihydroxy-acid dehydratase), and *ILV5* (ketol-acid reductoisomerase). In addition, proteins required for histidine biosynthesis (ATP phosphoribosyltransferase, histidine biosynthesis trifunctional protein, and imidazole glycerol phosphate synthase hisHF), leucine biosynthesis (pyruvate carboxyltransferase domain-containing protein), lysine biosynthesis (saccharopine dehydrogenase and alpha-aminoadipate reductase), and tryptophan biosynthesis (anthranilate synthase) were also found within downregulated clusters. Pathogenic fungi employ efficient strategies of nutrient assimilation to support cell growth and various virulence traits (including biofilm formation), and to combat environmental stresses.^80^–^82^ Amich and Bignell outlined a selection of fungal genes necessary in pathogenesis, such as *ILV, HIS, TRP*, and *LEU*, which encode the proteins mentioned earlier.^83^ Furthermore, the potential of amino acid synthesis/metabolism disruption as a novel antifungal target has been summarized^84^–^86^ For example, inhibition of the leucine biosynthetic enzyme isopropylmalate dehydrogenase can attenuate virulence in *Cryptococcus neoformans* and phytopathogenic *Magnaporthe* spp.,^84,87,88^ and methods of 3-isopropylmalate dehydratase inhibition for use in fungicides have even been patented.^84^ The most significantly decreased protein in response to SBC3 was 3-isopropylmalate dehydratase. These biosynthetic enzymes have proven attractive antifungal targets due to their absence in mammals, which must obtain essential amino acids from the diet.^86,89^

The ability of *Candida* spp. to adapt to hostile environments is a complex, multifactorial process dependent on adequate energy supplies for the maintenance of growth and metabolism.^90,91^ Components of cytochrome *c* (cytochrome *b–c1* complex subunit 7, cytochrome *b–c1* complex subunit Rieske, cytochrome *c* domain-containing protein encoded by *CYC1*) and a cytochrome *c* oxidase (COX) subunit, encoded by *COX4*, were decreased in abundance. The latter protein catalyses the final step of the electron transport chain and regulates oxidative phosphorylation for ATP synthesis.^92^–^94^ Furthermore, SBC3 significantly altered the abundance of NADH dehydrogenase [ubiquinone] flavoprotein 1 for NADH electron transfer to the respiratory chain (–25-fold). Other mitochondrial proteins significantly decreased in abundance are listed in Table S1.

Mitochondrial function extends beyond energy production to involvement in virulence and resistance mechanisms.^95,96^ Defective ATP-generating components of the mitochondria are capable of inhibiting *C. albicans*’s adherence and biofilm formation,^97^ while reduced respiration in phenazine-treated *C. albicans* achieved similar results.^98^ SBC3 (15 and 25 μg/ml) decreased Candida_ALS_N domain-containing protein (encoded by *CPAR2_404800*) by 9- and 5-fold, respectively (Supplementary File 1). Agglutinin-like sequence (Als) proteins are a well-established family of adhesins which initiate microbial colonization to biotic and abiotic surfaces.^99^ Pre-treatment of *C. parapsilosis* with SBC3 reduced yeast adherence to BECs by up to 92%, complementing previous studies by Bertini *et al*., which showed over 60% reduced adhesion to BECs in *C. parapsilosis CPAR2_404800* null mutants.^100^ SBC3 exposure can also reduce biofilm formation, which is an important virulence factor of *C. parapsilosis*.^101^ Biofilm visualization by SEM revealed a decreased presence of ECM in SBC3-treated cells. Comprised of polysaccharides, lipids, DNA, and proteins, ECM aids cell adhesion, offers an impenetrable barrier against antifungal agents and a nutrient source to cells.^102,103^ Furthermore, SBC3 appeared to hinder the transition from yeast to pseudohyphal forms. A number of genes are expressed during this shift, including many cell adhesion proteins, and are likely to explain the reduced expression of Candida_ALS_N domain-containing protein.^104^ Adherence to host tissue and biofilm formation are essential for the initial stages of infection. The ability of SBC3 to reduce these processes along with morphological shifts would adversely affect the ability of *C. parapsilosis* to colonize and disseminate in the host.

LFQ MS offers a powerful approach to studying cell proteomes and permit the identification of changes within complex sets of proteins and associated networks/pathways. Therefore, it has been applied to provide insight on the effect(s) of novel and conventional antifungal treatments.^105^–^108^ The results presented here indicate that exposure of *C. parapsilosis* to SBC3 results in reductions in proteins associated with protein synthesis, amino acid synthesis, and respiration and in virulence as measured by the inhibition of adherence and biofilm formation. These findings provide a novel insight into the mode of action of SBC3 and encourage further research into this interesting antifungal agent.

## Experimental section

### *Candida parapsilosis* culture conditions

*Candida parapsilosis* was cultured for 24–48 h in YEPD medium [2% (w/v) glucose (Sigma), 2% (w/v) peptone (Sigma-Aldrich), and 1% (w/v) yeast extract (Fisher)] at 30°C in an orbital shaker at 120 rpm. Stocks were kept on YEPD agar [as described with the addition of 2% (w/v) agar (Fisher)].

### SBC3 complex synthesis

SBC3 was synthesized as previously described.^47^

### SBC3 toxicity evaluation

SBC3 was dissolved in YEPD and 5% DMSO (Merck) and serially diluted in 96-well plates (Corning) to final concentrations of 0.49–125 μg/ml. Aliquots (100 μl) of *C. parapsilosis* cultures grown overnight were diluted in sterile phosphate buffered saline (PBS) and added to serially diluted complexes to obtain a final optical density (OD) of 0.05 (representing ∼ 4.5×10^5^ cells/ml). Plates were incubated at 30°C for 24 h in a static incubator. Growth was measured in a plate reader (Bio-Tek Synergy HT) at 600 nm.

### *Candida parapsilosis* protein extraction and purification

Sterile YEPD media supplemented with SBC3 (15 and 25 µg/ml) was inoculated with *C. parapsilosis*, and cells were grown for 8 h at 30°C in an orbital shaker at 120 rpm (*n* = 3, per sample group). Cells were washed three times in sterile PBS, resuspended in lysis buffer (pH 8.0) containing 6 M urea (Sigma), 2 M thiourea (Sigma-Aldrich), 0.1 M Tris-HCl (Sigma) and a range of protease inhibitors [10 μg/ml aprotinin (Caymen Chemical Company), leupeptin (Thermo Scientific), pepstatin A (Sigma), Tosyllysine Chloromethyl Ketone hydrochloride (TLCK), and 1 mM/ml phenylmethylsulfonyl fluoride (PMSF; Sigma)], and lysed via sonication. Cell debris was pelleted by centrifugation at 13 000 x g for 5 min. Proteins were acetone (Sigma) precipitated in a ratio of 1:3, sample to acetone, overnight at –20°C. The acetone was discarded, and proteins were resuspended in 25 µl of resuspension buffer (same as lysis buffer without protease inhibitors). Aliquots (2 µl) of samples were used to quantify proteins with the Qubit^TM^ quantification system (invitrogen). To the remaining samples, 105 μl of 50 mM ammonium bicarbonate and 1 μl 0.5 M dithiothreitol (Sigma-Aldrich) were added, and the samples were incubated at 56°C for 20 min. Samples were alkylated with 2.7 μl 0.5 M iodoacetamide (Sigma-Aldrich) and incubated in the dark at room temperature for 15 min. Proteins were digested with 1 μl 1% (w/v) ProteaseMAX and 1 μl 0.5 μg/μl Sequence Grade Trypsin (Promega) overnight at 37°C. Digestion was inhibited with 1 μl 100% trifluoroacetic acid (Sigma-Aldrich). Samples were incubated at room temperature for 5 min and centrifuged at 13 000 x g for 10 min. Peptides in the resulting supernatant were purified using C-18 spin columns (Pierce) and dried in a SpeedyVac concentrator (Thermo Scientific Savant DNA120) at 39°C for approximately 2 h. Samples were resuspended in 2% acetonitrile and 0.05% TFA, sonicated in a water bath for 5 min and centrifuged at 13 000 x g for 5 min. The supernatant was used for mass spectrometry analysis.

### Mass spectrometry

Purified samples (2 μl containing 0.75 μg protein) were loaded onto a Q Exactive Mass Spectrometer (ThermoFisher Scientific) using a 120-min reverse phase gradient as per previous methods.^109^ Raw MS/MS data were processed through the Andromeda search engine in MaxQuant software v.1.6.3.4 ^110^ using a *C. parapsilosis* database obtained from a UniProt-SWISS-PROT database to identify proteins (5777 entries, downloaded February 2021). Search parameters were followed as per previous methods.^111^

### 2,3,5-Triphenyltetrazolium chloride assay

*Candida parapsilosis* was grown in SBC3-supplemented media for 12 h at 30°C in an orbital shaker. Cells were harvested, washed twice with PBS, and resuspended accordingly to standardize the ODs of all samples to 0.15. A stock solution of TTC made in PBS was filter sterilized using a 0.22 μm pore filter and added to cell suspensions at a 1:10 dilution. The solutions were added to wells in a 96-well plate in 100 μl aliquots containing 0.5 mg/ml TTC per well and incubated for 8 h at 30°C in a static incubator. The supernatant was removed, and the remaining formazan crystals were solubilized with 100% DMSO for 15 min in an orbital shaker. The absorbance was measured at 550 nm.

### Adherence assay

*Candida parapsilosis* was grown in YEPD media containing 15, 25, 50, and 75 μg/ml SBC3 at 30°C for 24 h. Cells were harvested, resuspended in PBS, and enumerated. BECs were self-isolated by gently harvesting from the inside cheek of a healthy individual using a tongue depressor. Yeast cells and BECs were combined in a ratio of 50:1, yeast: BEC, in PBS to a final volume of 2 ml. The cell mixture was incubated at 30°C for 120 min at 120 rpm. The solution was passed through a polycarbonate membrane with 30 μm pores and washed with 8 ml of PBS to eliminate non-adhered *C. parapsilosis* cells. The remaining BECs with attached *C. parapsilosis* cells were applied to microscope glass slides that were dried overnight, heat fixed, and stained with 0.5% crystal violet dye (ACROS Organics) for 1 min. The average number of adhering yeast cells per BEC was determined after counting 100 BEC per treatment.

### Biofilm assay

*Candida parapsilosis* was cultured overnight, diluted in YEPD medium and aliquoted [90 μl of OD 0.1 (∼ 9×10^5^ cells/ml)] into flat-bottom 96-well plates (Corning). A range of SBC3 concentrations were added in 10 μl aliquots. Plates were incubated at 30°C for 48 h in a static incubator. Growth was measured as previously described. Planktonic cells were removed, and plates were washed four times with deionized water. Adhered cells were stained with 0.1% crystal violet dye for 15 min. The dye was removed, and plates were washed to remove excess. The remaining stain was air dried overnight and solubilized with 100 μl, 30% acetic acid (Fisher). The intensity of staining was measured at 550 nm.

### Scanning electron microscopy

*Candida parapsilosi*s cell suspensions (OD 0.1) treated with SBC3 were grown in 6-well plates containing glass slides for 48 h at 30°C in a static incubator. SBC3 solutions were made with DMSO and YEPD. Control samples consisted of YEPD and 0.375% DMSO as per treated samples. The supernatants were removed from wells and the remaining adhered cells on glass slides were fixed with 2.5% glutaraldehyde (Sigma) for 2 h. Cells were washed with pre-warmed PBS and dehydrated in increasing ethanol solutions (35%, 50%, 70%, 80%, 90%, and twice in 100%) for 10 min each. Slides were dried with 100% hexamethyldisilazane (Sigma-Aldrich) for 10 min followed by overnight air-drying. Slides were sputtered with gold (6–12 nm) and imaged on a scanning electron microscope (HITACHI S-3200N).

### Data analysis

Graphs were constructed and analysed on GraphPad Prism v.9.2.0. Statistical analysis via one-way ANOVA with Tukey's multiple comparisons test was performed on the means of all sample groups.

The resulting LFQ intensity values were processed through Perseus v.1.6.6.0 (www.maxquant.net/) to generate statistical and graphical analysis.^112^ Normalized LFQ intensity values were used as protein abundance measurements. Proteins were filtered to remove contaminants and peptides identified by site. LFQ intensities were log_2_ transformed, and samples were grouped accordingly (control, 15 μg/ml treatment, and 25 μg/ml treatment). Proteins not found in three out of the three replicates in at least one group were removed. The data were imputated to replace missing values with values that imitate signals of low abundance proteins randomly selected from a distribution designated by a downshift of 1.8 times the mean standard deviation of all measured values and a width of 0.3 times this standard deviation. Comparisons between two sample groups were conducted via pair-wise Student's *t*-tests with an FDR cut-off of 0.05 on post-imputated data. Volcano plots were produced by plotting log_2_ fold changes on the *x* axis and –log *P*-values on the *y* axis for each sample group comparison. SSDA proteins [ANOVA, *P* < 0.05, fold change ≥1.5] were used in subsequent analyses. LFQ intensities were *z*-score normalized for hierarchal clustering of the median expression values of SSDA proteins using Euclidean distance. A Fisher's Exact test (Benjamini–Hochberg, FDR cut-off of 5%) was used to perform GO term enrichment analysis of major protein clusters for enrichment in GO biological process, GO cellular component, and GO molecular function. The mass spectrometry proteomics data have been deposited to the ProteomeXchange Consortium via the PRIDE partner repository with the dataset identifier PXD025693.^113^

Cytoscape v.3.8.2 bioinformatics software was used to map SSDA proteins retrieved from UniProt gene lists in Perseus. A high confidence score (0.9) was used to generate interaction networks of up- and downregulated protein pathways for treatment versus control groups. Functional enrichment analysis was performed on protein clusters for enrichment in UniProt key words to identify and annotate functions/processes.

## Supplementary Material

mfac046_Supplemental_Files

## Data Availability

The mass spectrometry proteomics data have been deposited to the ProteomeXchange Consortium via the PRIDE partner repository with the dataset identifier PXD025693.^113^
