# Supplementary material for: Exposure of Candida parapsilosis to the silver(I) compound SBC3 induces alterations in the proteome and reduced virulence
Source: Metallomics. 2022 Jun 25;14(8):mfac046. doi: 10.1093/mtomcs/mfac046 (PMC9348618; doi:10.1093/mtomcs/mfac046)
Supplement: mfac046_Supplemental_Files [file mfac046_supplemental_files.zip › Legends_to_Supplementary_Figures.docx]

Legends to Supplementary Figures

**Figure S1.** Growth curve of *C. parapsilosis* with SBC3 (15 and 25 μg/ml). YEPD supplemented with SBC3 was inoculated with *C. parapsilosis* and incubated for up to 12 h at 30°C in an orbital shaker at 120 rpm. Readings were taken at various time intervals using a spectrophotometer at an optical density (OD) of 600 nm to determine fungal growth. All values are the mean ± S.E, n = 3.

**Figure S2.** Cytoscape network analysis of differently expressed proteins in *C. parapsilosis* treated with SBC3 15 μg/ml versus control, 25 μg/ml versus control and 25 μg/ml versus 15 μg/ml SBC3. SSDA proteins were uploaded onto Cytoscape software using a high confidence score (0.9) to create interaction networks. Nodes represent individual proteins that are interconnected to establish pathways. Differential expression is highlighted in red (increased abundance) and blue (decreased abundance). Functions associated with protein clusters are annotated according to functional enrichment in UniProt key words.

**Figure S3.** Microscopy image of *C. parapsilosis* cells attached to a buccal epithelial cell (BEC).
