# Supplementary material for: Exposure of Candida parapsilosis to the silver(I) compound SBC3 induces alterations in the proteome and reduced virulence
Source: Metallomics. 2022 Jun 25;14(8):mfac046. doi: 10.1093/mtomcs/mfac046 (PMC9348618; doi:10.1093/mtomcs/mfac046)
Supplement: mfac046_Supplemental_Files [file mfac046_supplemental_files.zip › Supplementary_Table_1.docx]

| **SBC3 15 μg/ml** | | | | | |
| --- | --- | --- | --- | --- | --- |
| **Protein name** | **Gene name** | **Peptides** | **Sequence coverage [%]** | **Score** | **Fold change** |
| Chitinase | CPAR2_502120 | 16 | 24.3 | 115.93 | +222.2 |
| CFEM2 | CPAR2_402910 | 1 | 6 | 14.536 | +207.2 |
| CFEM5; CFEM6 | CPAR2_300120; CPAR2_300110 | 4 | 8.7 | 116.31 | +162.5 |
| Uncharacterized protein | CPAR2_304370 | 39 | 35.2 | 102.48 | +42.5 |
| Uncharacterized protein | FET3 | 13 | 26.6 | 129.09 | +35.0 |
| RRM domain-containing protein | CPAR2_100830 | 13 | 62.6 | 80.232 | +35.0 |
| Thioredoxin domain-containing protein | CPAR2_402640 | 12 | 87.4 | 115.02 | +29.8 |
| Uncharacterized protein | CDR1 | 39 | 34.7 | 323.31 | +25.0 |
| Uncharacterized protein | CPAR2_303120 | 8 | 9.6 | 63.535 | +22.8 |
| Chitinase | CPAR2_502130 | 7 | 15.3 | 34.261 | +21.1 |
| Peptidylprolyl isomerase | CPAR2_202630 | 5 | 55.3 | 26.502 | +20.3 |
| Uncharacterized protein | CPAR2_208430 | 9 | 51 | 323.31 | +19.6 |
| FAD-binding FR-type domain-containing protein | CFL5 | 11 | 19.5 | 56.965 | +18.3 |
| Uncharacterized protein | SCR2; ADH; SCR3 | 12 | 59.9 | 112.28 | +17.7 |
| FAD-binding FR-type domain-containing protein | CPAR2_210110; CPAR2_808110 | 9 | 15.4 | 28.301 | +17.2 |
| Pyr_redox_2 domain-containing protein | CPAR2_802640 | 11 | 33.3 | 57.834 | +14.1 |
| Uncharacterized protein | CPAR2_603800 | 38 | 30.8 | 25.22 | +11.9 |
| Uncharacterized protein | CPAR2_301610 | 7 | 19.4 | 59.647 | +11.4 |
| Alpha-mann_mid domain-containing protein | CPAR2_500360 | 11 | 16.3 | 31.539 | +10.1 |
| Phosphatidylinositol transfer protein SFH5 | CPAR2_804790 | 9 | 37.2 | 22.669 | +7.4 |
| AMP-binding domain-containing protein | CPAR2_600460 | 8 | 26.8 | 76.429 | -5.4 |
| MFS domain-containing protein | CPAR2_104450 | 4 | 15.4 | 29.788 | -5.5 |
| Uncharacterized protein | CPAR2_701510 | 9 | 35.9 | 34.579 | -5.6 |
| Uncharacterized protein  Mitochondrial import inner membrane translocase subunit TIM44 | CPAR2_504060 | 15 | 41.8 | 58.004 | -5.9 |
| Uncharacterized protein | CPAR2_102890 | 2 | 20 | 14.493 | -5.9 |
| NAD(P)-bd_dom domain-containing protein | CPAR2_503600 | 16 | 60.2 | 126.83 | -6.0 |
| Uncharacterized protein | CPAR2_807040 | 4 | 29.9 | 27.563 | -6.2 |
| Beta_elim_lyase domain-containing protein | CPAR2_203970 | 9 | 48.2 | 34.776 | -6.3 |
| Threonine dehydratase | ILV1 | 13 | 32.8 | 50.422 | -6.6 |
| Oxidored_q6 domain-containing protein | CPAR2_808860 | 3 | 16.4 | 11.678 | -7.0 |
| Cytochrome b5 heme-binding domain-containing protein | CPAR2_703300 | 2 | 18 | 7.4867 | -7.1 |
| Uncharacterized protein | CPAR2_800850 | 6 | 38.8 | 22.069 | -7.5 |
| Uncharacterized protein | ILV3 | 16 | 57.4 | 147.15 | -8.1 |
| Uncharacterized protein | CPAR2_407280 | 2 | 14.2 | 16.392 | -8.4 |
| Candida_ALS_N domain-containing protein | ALS7; CPAR2_404780; ALS3 | 7 | 10.6 | 54.262 | -8.6 |
| Uncharacterized protein | CPAR2_503690 | 15 | 51.1 | 72.504 | -10.0 |
| Uncharacterized protein | CPAR2_206380 | 29 | 22.8 | 83.333 | -12.7 |
| Glutamine amidotransferase type-2 domain-containing protein | CPAR2_206810 | 32 | 24.2 | 115.55 | -13.1 |
| 3-isopropylmalate dehydratase | CPAR2_800360 | 26 | 40 | 112.53 | -15.5 |

| **SBC3 25 μg/ml** | | | | | |
| --- | --- | --- | --- | --- | --- |
| **Protein name** | **Gene name** | **Peptides** | **Sequence coverage [%]** | **Score** | **Fold change** |
| CFEM2 | CPAR2_402910 | 1 | 6 | 14.536 | +178.5 |
| CFEM6; CFEM5 | CPAR2_300120;  CPAR2_300110 | 4 | 8.7 | 116.31 | +171.6 |
| Chitinase | CPAR2_502120 | 16 | 24.3 | 115.93 | +139.2 |
| Uncharacterized protein | CPAR2_304370 | 39 | 35.2 | 102.48 | +57.5 |
| RRM domain-containing protein | CPAR2_100830 | 13 | 62.6 | 80.232 | +42.7 |
| Uncharacterized protein | CDR1 | 39 | 34.7 | 323.31 | +32.6 |
| Thioredoxin domain-containing protein | CPAR2_402640 | 12 | 87.4 | 115.02 | +27.8 |
| Uncharacterized protein | FET3; CPAR2_603590 | 13 | 26.6 | 129.09 | +22.2 |
| Uncharacterized protein | SCR2; ADH; SCR3 | 12 | 59.9 | 112.28 | +20.8 |
| Thioredoxin domain-containing protein | CPAR2_403210 | 6 | 67.8 | 22.101 | +18.8 |
| Uncharacterized protein | CPAR2_603800 | 38 | 30.8 | 25.22 | +18.1 |
| Pyr_redox_2 domain-containing protein | CPAR2_802640 | 11 | 33.3 | 57.834 | +17.3 |
| CFEM7 | PAR2_500080 | 2 | 33.8 | 22.019 | +16.0 |
| Chitinase | CPAR2_502130 | 7 | 15.3 | 34.261 | +15.5 |
| Uncharacterized protein | CPAR2_208430 | 9 | 51 | 323.31 | +15.0 |
| Uncharacterized protein | CPAR2_209770 | 7 | 7.1 | 34.804 | +14.6 |
| Uncharacterized protein | CPAR2_206560 | 4 | 31.2 | 30.568 | +13.7 |
| Uncharacterized protein | CPAR2_301610 | 7 | 19.4 | 59.647 | +13.5 |
| Uncharacterized protein | CPAR2_303120 | 8 | 9.6 | 63.535 | +13.5 |
| PKS_ER domain-containing protein | CPAR2_211280; CPAR2_211230 | 13 | 58.3 | 79.107 | +13.0 |
| Uncharacterized protein | CPAR2_400800 | 4 | 55.9 | 11.006 | -7.1 |
| Uncharacterized protein | CPAR2_108260 | 17 | 56.3 | 105.03 | -7.1 |
| Uncharacterized protein | CPAR2_200690 | 30 | 57.8 | 318.26 | -7.2 |
| H/ACA ribonucleoprotein complex subunit NOP10 | CPAR2_701770 | 4 | 62.7 | 121.06 | -7.2 |
| Squalene monooxygenase | CPAR2_210480 | 10 | 24.9 | 38.203 | -7.4 |
| MFS domain-containing protein | CPAR2_600460 | 8 | 26.8 | 76.429 | -7.8 |
| DNA-directed RNA polymerase subunit | RPA190 | 26 | 26.2 | 124.03 | -7.8 |
| Complex1_49kDa domain-containing protein | CPAR2_703410 | 16 | 43.3 | 144.06 | -8.2 |
| Uncharacterized protein | CPAR2_407940 | 39 | 20.7 | 150.2 | -8.3 |
| Mitochondrial import inner membrane translocase subunit TIM44 | CPAR2_504060 | 15 | 41.8 | 58.004 | -8.7 |
| Acetolactate synthase | ILV2 | 25 | 44.8 | 190.6 | -9.0 |
| Glutamine amidotransferase type-2 domain-containing protein | CPAR2_206810 | 32 | 24.2 | 115.55 | -9.7 |
| zf-CHCC domain-containing protein | CPAR2_405230 | 10 | 71.8 | 58.2 | -10.0 |
| NAD(P)-bd_dom domain-containing protein | CPAR2_503600 | 16 | 60.2 | 126.83 | -11.0 |
| 3-isopropylmalate dehydratase | CPAR2_800360 | 26 | 40 | 112.53 | -15.6 |
| Uncharacterized protein | CPAR2_108760 | 24 | 44.7 | 323.31 | -16.1 |
| Uncharacterized protein | ILV3 | 16 | 57.4 | 147.15 | -16.4 |
| Uncharacterized protein | CPAR2_601420 | 5 | 47 | 127.71 | -20.9 |
| NADH dehydrogenase [ubiquinone] flavoprotein 1, mitochondrial | CPAR2_103910 | 21 | 64 | 99.345 | -24.7 |
| Ribosomal protein L37 | CPAR2_207910 | 3 | 26.7 | 10.947 | -48.6 |
