# Supplementary figures and images for: Exposure of Candida parapsilosis to the silver(I) compound SBC3 induces alterations in the proteome and reduced virulence
Source: Metallomics. 2022 Jun 25;14(8):mfac046. doi: 10.1093/mtomcs/mfac046 (PMC9348618; doi:10.1093/mtomcs/mfac046)

## Slide 1
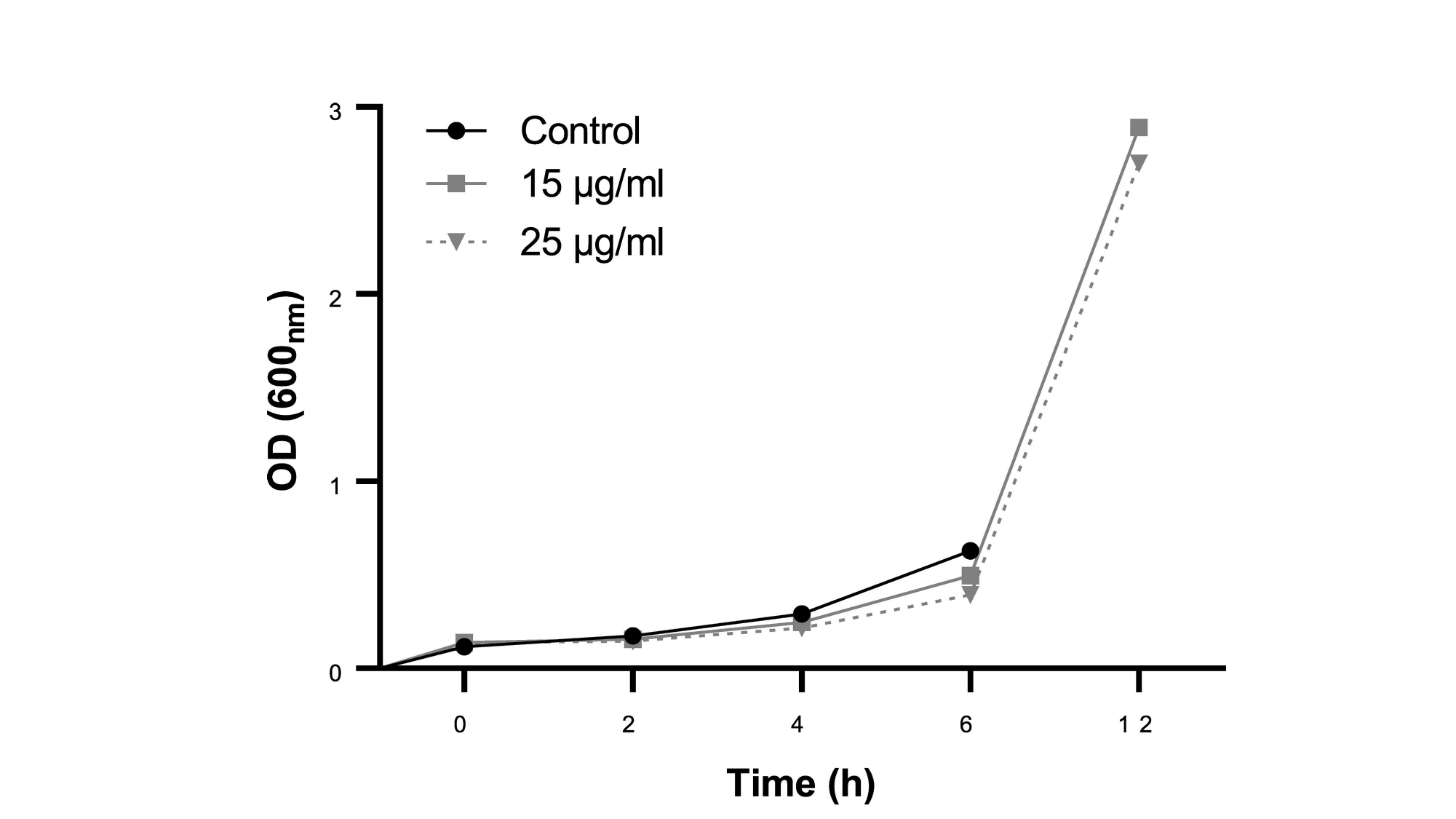

Supplement: mfac046_Supplemental_Files [file mfac046_supplemental_files.zip › Supp_Fig_1.pptx]

## Slide 1
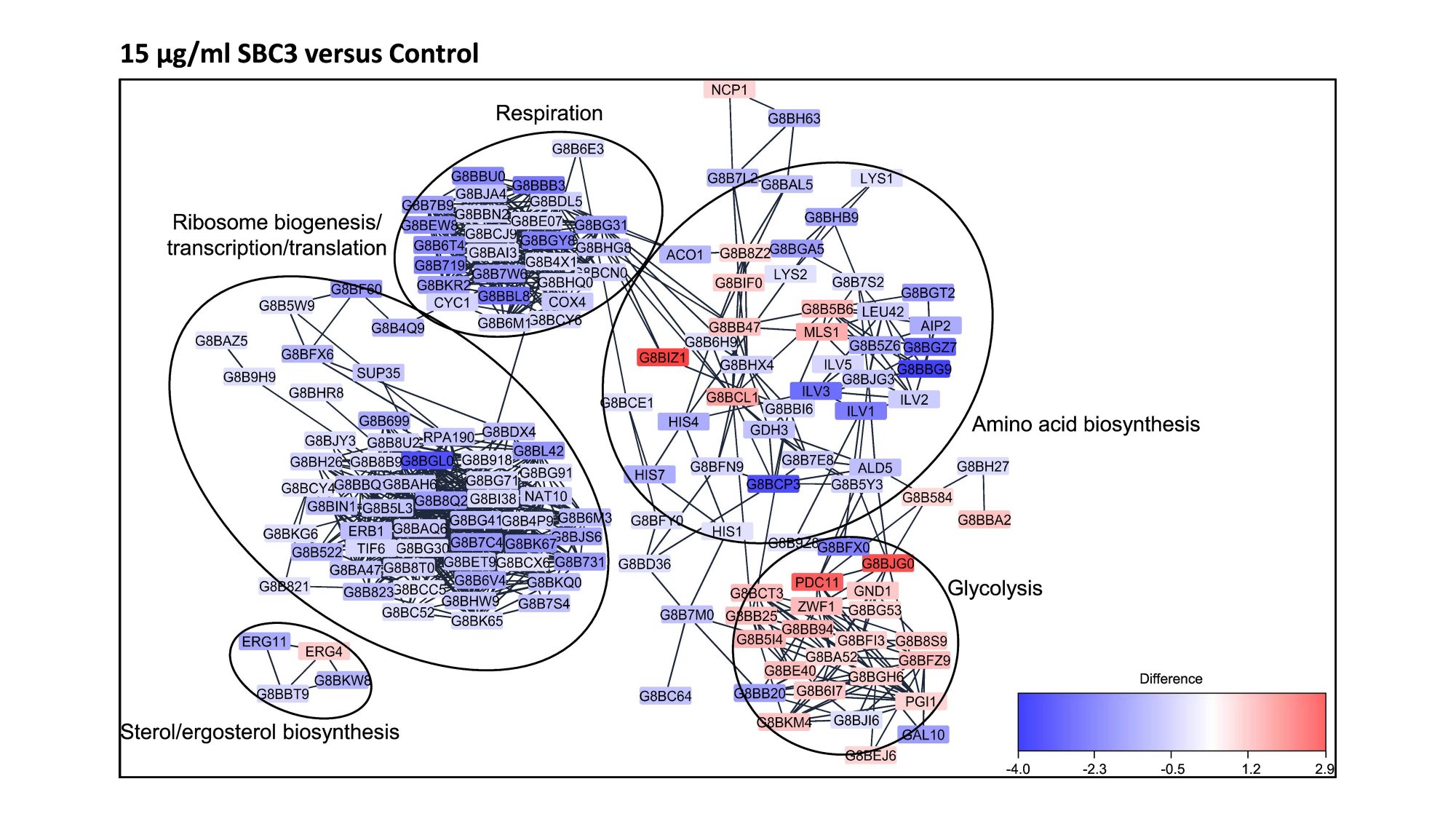

## Slide 2
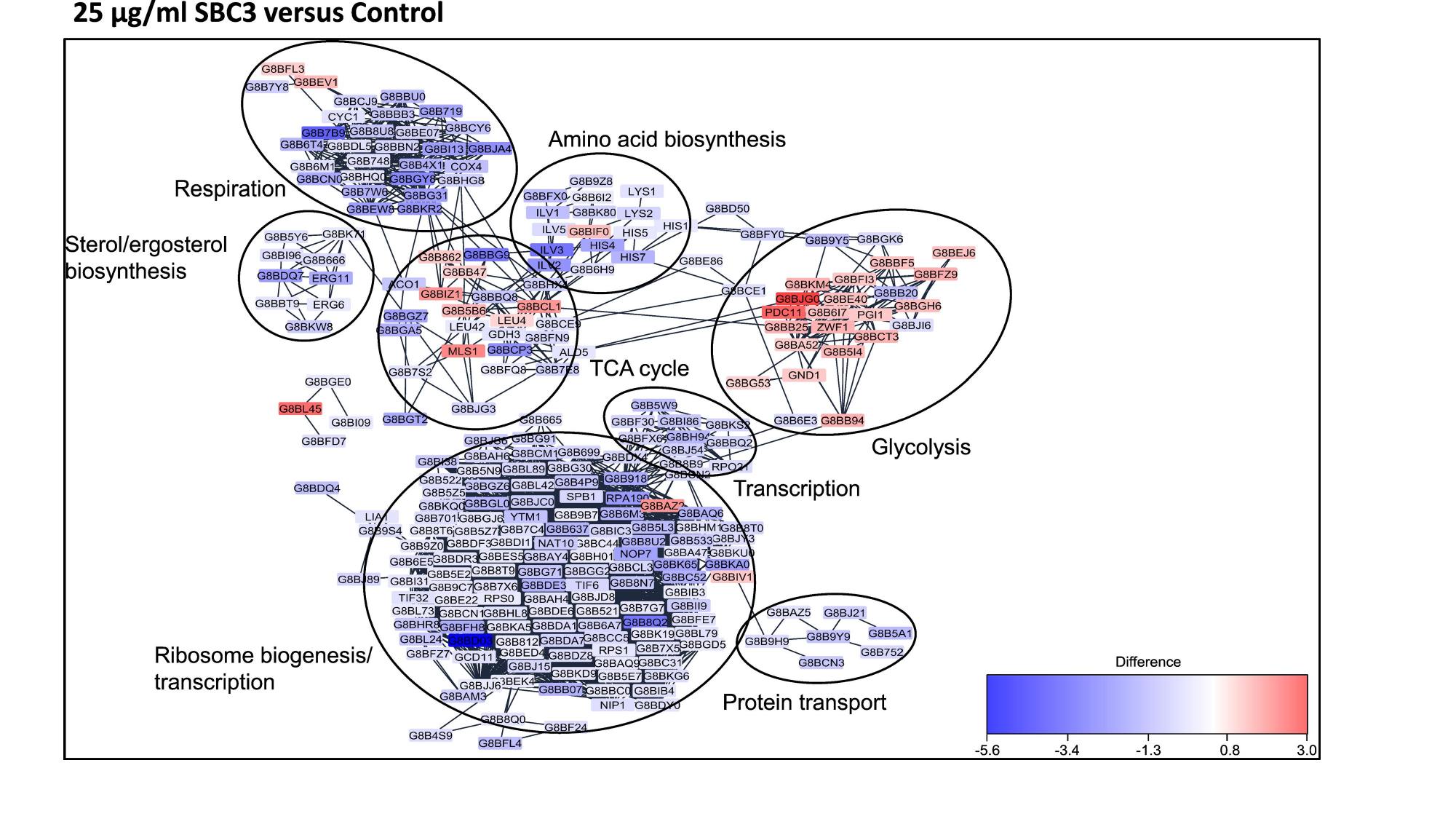

## Slide 3
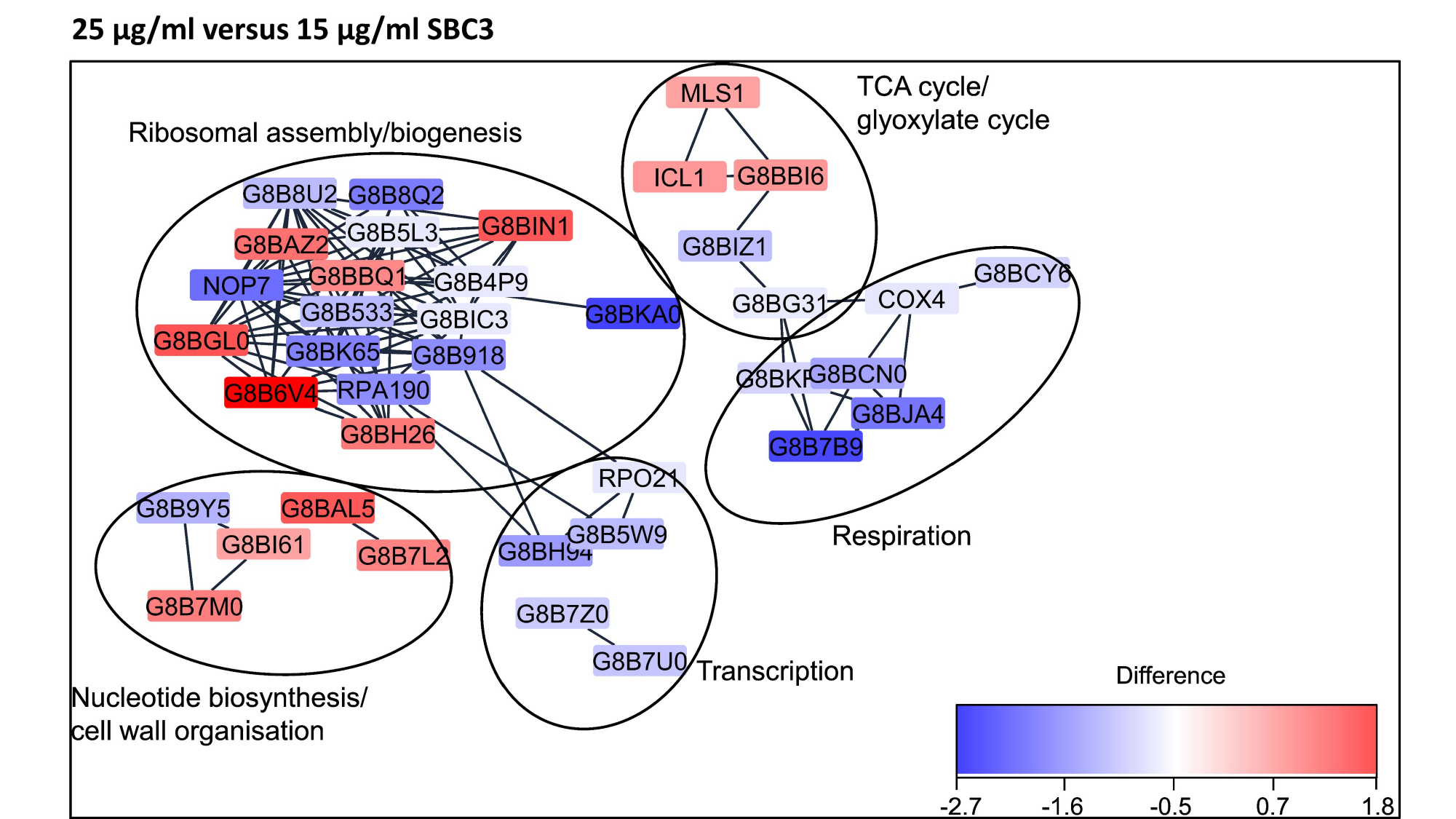

Supplement: mfac046_Supplemental_Files [file mfac046_supplemental_files.zip › Supp_Fig_2.pptx]

## Slide 1
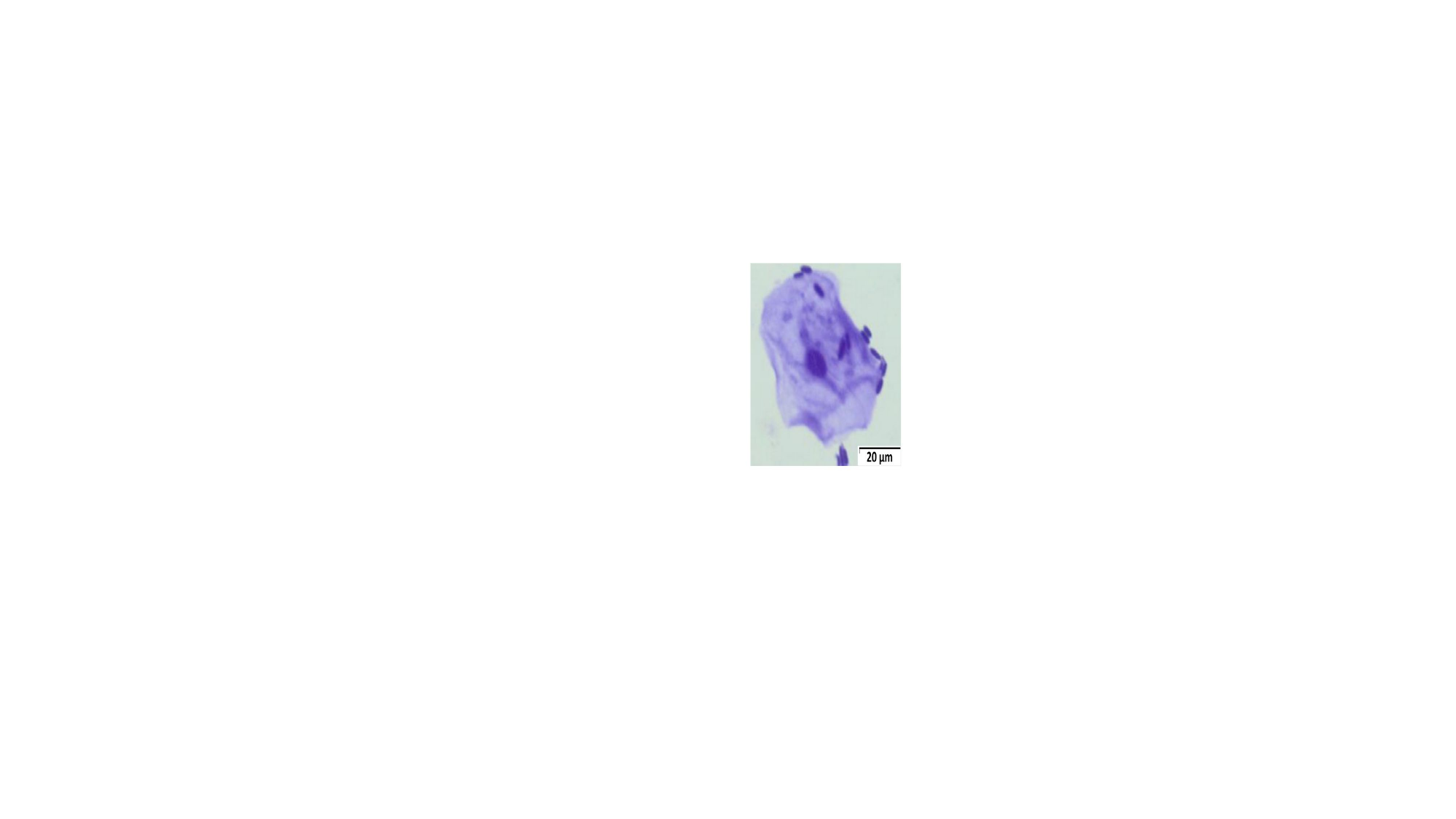

Supplement: mfac046_Supplemental_Files [file mfac046_supplemental_files.zip › Supp_Fig_3.pptx]
